# Defining Two Chemosensory Arrays in *Shewanella oneidensis*

**DOI:** 10.3390/biom13010021

**Published:** 2022-12-22

**Authors:** Emma M. Fortier, Sophie Bouillet, Pascale Infossi, Amine Ali Chaouche, Leon Espinosa, Marie-Thérèse Giudici-Orticoni, Emilia M. F. Mauriello, Chantal Iobbi-Nivol

**Affiliations:** 1 Laboratoire de Bioénergétique et Ingénierie des Protéines, Institut de Microbiologie de la Méditerranée, Centre National de la Recherche Scientifique, Aix-Marseille Université, 13402 Marseille, France; 2 NIH, NCI, 37 Convent Drive, Bethesda, MD 20814, USA; 3 Laboratoire de Chimie Bactérienne, Institut de Microbiologie de la Méditerranée, Centre National de la Recherche Scientifique, Aix-Marseille Université, 13402 Marseille, France

**Keywords:** chemosensory systems, chemoreceptors, *Shewanella oneidensis*

## Abstract

*Shewanella oneidensis* has 2 functional chemosensory systems named Che1 and Che3, and 27 chemoreceptors. Che3 is dedicated to chemotaxis while Che1 could be involved in RpoS post-translational regulation. In this study, we have shown that two chemoreceptors Aer2so and McpAso, genetically related to the Che1 system, form distinct core-signaling units and signal to Che1 and Che3, respectively. Moreover, we observed that Aer2so is a cytoplasmic dynamic chemoreceptor that, when in complex with CheA1 and CheW1, localizes at the two poles and the centre of the cells. Altogether, the results obtained indicate that Che1 and Che3 systems are interconnected by these two chemoreceptors allowing a global response for bacterial survival.

## 1. Introduction

Chemosensory systems allow a rapid and adaptive response to bacterial environmental cues by activating directed swimming or metabolism reorientation [1]. Indeed, chemosensory systems are involved in chemotaxis allowing bacteria to move toward attractants or away from repellents but also in biofilm formation or virulence. It has been shown in *Escherichia coli* that to transduce external signals and induce an oriented swim, CheA, a histidine kinase; CheW, a connecting protein; and methyl-accepting chemotaxis proteins (MCPs), also named chemoreceptors, are required. Altogether, these proteins constitute core-signaling units with various stoichiometries: 6MCPs:2CheA:2CheW in *E. coli* [2,3,4], 44MCPs:2CheA:22CheW in *Sinorhizobium meliloti* [5], and a CheA dimer-CheW stoichiometry of 1:14 in *Vibrio cholera* [6]. These complexes are localized at the cell pole forming chemosensory arrays classified as F7 arrays according to the chemotaxis system class evolution defined by Wuichet and Zhulin [7,8]. Upon changes in concentration of attractants or repellents, a conformational change is induced in the MCPs leading to an autophosphorylation of CheA. The latter phosphorylates CheY, a response regulator that, in its phosphorylated form, interacts with FliM, the switch protein of the flagellum rotor, leading to a change in the flagellar rotation termed tumble. The biased tumbling frequencies lead to directed bacterial motility [2,9]. While *E. coli* synthetizes 5 MCPs and 1 F7 system dedicated to chemotaxis, *V. cholerae* and *Pseudomonas aeruginosa* have 43 and 26 chemoreceptors and 3 and 4 chemosensory pathways, respectively [10,11]. It was established that multiple MCPs can converge to the same Che pathway leading to the integration of several signals for the same cellular response [12,13]. In addition, the presence of multiple systems allows bacteria to sense a large variety of signals and cope with versatile and harsh environments [7,14]. If multiple MCPs can signal to one Che system, only MCPs where the conserved C terminal region have the same length, measured as the number of heptades [15,16], can form heterotrimers of homodimers and make networks with the same CheA and CheW proteins. A heptad is a repeated seven-amino acid sequence constituting the MCP signaling domain and was firstly established for transmembrane MCPs [15]. The five MCPs of *E. coli* all belong to the 36H class.

*V. cholerae* and *P. aeruginosa* bear two types of chemosensory pathways that are phylogenetically related in the two species [17] and each belong to the F6 or F7 class. The F7 pathways functionally diverge from that of *E. coli* as they evolved towards still unknown physiological functions. Instead, the F6 class is involved in chemotaxis in these organisms. Chemoreceptor arrays were visualized by CryoET in various organisms [18,19,20]. It has been shown that F7 arrays are longer than F6 arrays often associated with 40H class MCPs, suggesting that they are composed of MCPs of different classes [18].

**Figure 1:**
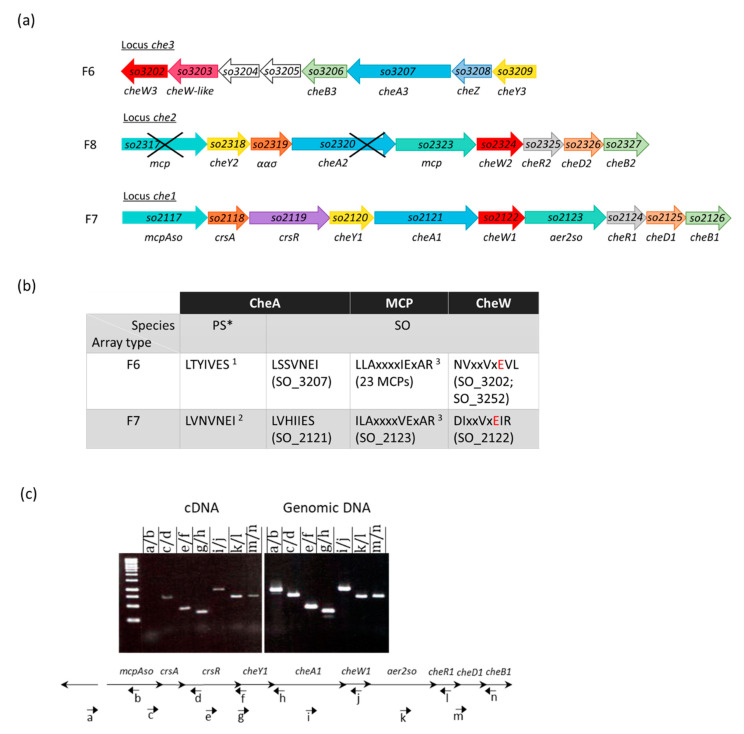
Chemosensory systems in *S. oneidensis*. (**a**) Gene clusters of *S. oneidensis* MR1 encoding the three chemosensory systems. Black crosses in *che2* locus indicate the presence specific to this strain of insertion sequences; (**b**) consensus motifs found in CheA, CheW, and MCPs in *S. oneidensis* (SO). Motifs were defined based on motifs defined in *Pseudomonas species* [17] (PS *): ^1^ from the motif “LTYIVES” established in F6 CheA sequences, ^2^ from the motif “LVNVNEI” established in F7 CheA sequences, and ^3^ same motif as defined in *Pseudomonas species*. Red cases, highly conserved glutamate in CheW sequences; (**c**) analysis of *che1* transcription by RT-PCR. Black small arrows and lowercase stand for the direction and name of primers. The PCR experiments were carried out using either cDNA or genomic DNA as a template and with different couples of primers, as indicated below the gel. The cDNA was obtained by the reverse transcription of RNA extracted from *S. oneidensis* cells grown in aerobiosis and sampled during the exponential phase. The amplification products were submitted to a 1.2% agarose gel electrophoresis. A DNA ladder (O’GeneRuler 1kb, Thermo Scientific) was used as a reference (left lane).

*Shewanella oneidensis* is a bacterium found in salted and fresh water as well as in sediments. To cope with harsh and versatile conditions found in such biotopes, *S. oneidensis* developed original and adapted respiration mechanisms, and systems allowing resistance to toxic compounds [21]. *S. oneidensis* genome encodes three Che clusters (Figure 1a). The Che3 machinery is the best characterized (Figure S1). It contains eight genes (*so_3202-so_3209*), forms F6 arrays, is responsible for chemotaxis, and is involved in solid-surface-associated and floating-biofilm formation [22,23]. Inactivation of *cheA3*, *cheW3*, or *cheY3* leads to the lack of chemotaxis in *S. oneidensis* [22,24]. The *che2* locus (*so_2317* to *so_2327*) contains nine genes encoding six Che proteins CheA2, CheY2, CheW2, CheR2, CheD2, and CheB2; two MCPs (*so_2317* and *so_2321*); and an uncharacterized anti-sigma factor antagonist. However, *so_2317*, the first gene of the locus, and the *cheA2* gene are interrupted by the insertion of two *tnpA* (transposase), very often found on the *S. oneidensis* MR1-R genome suggesting that Che2 is incomplete or not transcribed [25]. It is noteworthy that in other *Shewanella* species like *S. decolorationis* LDS1, the *che2* locus is not disrupted [24,26]. The *che1* locus encompasses 10 genes: 6 genes encoding Che proteins, namely CheA1, CheY1, CheW1, CheR1, CheD1, and CheB1; 2 genes encoding MCPs, SO_2117 and SO_2123; and 2 genes coding for CrsR, a regulator with an anti-sigma factor domain and CrsA, an anti-sigma factor antagonist [27]. CrsR and CrsA are involved in the posttranslational regulation of RpoS by forming a partner switching system that controls the RpoS availability according to the environmental conditions [28,29] (Figure S1). *S. oneidensis* genome also bears 24 orphan genes coding for MCPs.

Here, based on what is known in *P. aeruginosa*, we have bioinformatically characterized *S. oneidensis* MCPs and Che systems and attributed each MCP to a Che pathway. We have also determined the function of some chemoreceptors in *S. oneidensis* and developed a strategy to biochemically determine the components of chemosensory arrays. Finally, by microscopy we have unveiled that the MCP SO_2123 forms dynamic complexes unevenly distributed in the cell and that contrarily to its homologs in other bacteria, *S. oneidensis* Che1 presents multiple localization patterns in cells.

## 2. Materials and Methods

### 2.1. Bacterial Strains and Culture Conditions

All strains used in this study are listed in the Table 1. Strains were routinely grown aerobically at 28 °C (*S. oneidensis* strains) or at 37 °C (*E. coli* strains) in Lysogeny Broth (LB) medium or on LB agar (17 g/L) [30]. When appropriated, antibiotics were used at the following concentrations: rifampicin (10 μg/mL) or chloramphenicol (25 μg/mL). All *S. oneidensis* strains are derivatives of the MR1-R strain referred as WT. C600 *E.coli* p*M2-6his* cells were grown at 28 °C in LB medium containing 0.5 μg mL^−1^ thiamine and 25 μg mL^−1^ 5-aminolevulinic acid (Sigma-Aldrich, Saint-Quentin-Fallavier, France), and induced at an OD_600nm_ of 0.8 for 2 h with 1 mM IPTG. BT3388 *E. coli* and *S. oneidensis* cells were grown at 28 °C in M63 minimum medium supplemented with 1 mM MgCl_2_ and 0.5% casa-amino acids and induced at an OD_600nm_ of 0.8 overnight with 0.1 mM IPTG.

### 2.2. Construction of Deletion Mutants

Deletions of *so_2122* (*cheW1*) and *so_2117* (*mcpAso*) were performed by cloning two 500 bp fragments flanking the gene to be deleted into the pKNG101 suicide vector [33] at the SalI and SpeI (*so_2122*), and at SpeI and BamHI (*so_2117*) restriction sites as described before [32]. The resulting plasmids were transformed into *E. coli* CC118λpir and then transferred to MR1-R wild type (single deletion) by conjugation with the *E. coli* helper strain 1047/pRK2013 [37]. The plasmid was integrated into the chromosome by a first recombination event. The plasmid was then removed from the chromosome by a second recombination event by adding 6% of sucrose in LB agar medium. Clones were tested by PCR to select those for which the recombination event led to gene deletion. The deletions include all the coding sequence (from start to stop codons) of the genes of interest. Deletion of the target genes was confirmed by sequencing the appropriate overlapping region.

### 2.3. Plasmid Construction

All plasmids used in this study are listed in Table 2. To construct p*M2-6his* and p*AWM2-6his* plasmids, the coding sequences were PCR-amplified using chromosomal *S. oneidensis* DNA as thetemplate, primers containing appropriate restriction sites were used, as well as the original Shine Dalgarno sequence to maintain the correct ratio of each protein when several genes are cloned. All PCR amplifications were performed with Q5 DNA polymerase (New England Biolabs, Evry, France). For fluorescence experiments, the primers contained a common spanner sequence containing ggaagtggaggaagtgga at its 5′ or 3′-ends. The gene coding GFP was amplified with complementary spanner sequence at the 5′-ends for p*M2-GFP* and p*mcpAso-GFP*, and 3′-ends for p*GFP-AWM2*. Chimeric sequences were constructed by a second PCR by mixing an equimolar amount of the corresponding PCR products and convergent primers. The chimeric gene sequences were cloned between two restriction sites of the p33Tac vector. The resulting plasmids were introduced into strain C600 and controlled by DNA sequencing before conjugation in *S. oneidensis* strains. All constructions were checked by DNA sequencing using appropriate primers.

### 2.4. RT-PCR Experiments

#### 2.4.1. RNA Extraction

*S. oneidensis* was aerobically grown in LB at 28 °C. A 1 mL-sample was taken during the exponential phase (OD600 ≈ 2) and an equal volume of RNAlater (Thermo Fisher Scientific, USA) was added to the cells. The bacterial suspension was incubated for 1 h at room temperature and then centrifuged for 5 min at 8000 rpm. The cell pellet was immediately frozen and conserved at −80 °C. RNA was extracted from the cells using the Maxwell instrument and the associated RNA purification kit (Promega, Charbonnières-les-Bains, France). The preparation was then treated with Turbo DNase (Thermo Fisher Scientific, USA) to avoid DNA contamination. The quality and integrity of the RNA preparation were checked by using the Experion automated electrophoresis system (Biorad, Gemenos, France).

#### 2.4.2. cDNA Synthesis

Reverse transcription was performed with 1 μg of RNA using the GoScript Reverse Transcriptase (Promega, Charbonnières-les-Bains, France), random hexameric primers in a buffer containing 2.5 mM MgCl_2_.

#### 2.4.3. PCR Amplification

Amplifications were carried out using either cDNA synthetized as indicated above or genomic DNA from *S. oneidensis* as a template, and the Q5^®^ High-Fidelity DNA Polymerase (NEB). The cDNA was diluted at 1/25 before use. The samples were submitted to 30 cycles consisting of 10 s at 98 °C, 10 s at 55 °C, and 30 s at 72 °C.

#### 2.4.4. Cell Fractionation and Preparation of Protein Extracts

Cells (4g) were resuspended in 5 mL of Tris-HCl (40 mM pH 7.4) buffer and one tablet of protease inhibitors (cOmplete ULTRA Tablets, Roche) was added. The cell suspension was disrupted by French press (twice at 700 PSI). Unbroken cells were removed by centrifugation at 5000 rpm for 10 min and the supernatant was ultracentrifuged at 50,000 rpm in an OPTIMA Max XP, rotor TLA 110, Beckman for 1 h at 4 °C to separate the soluble and membrane fraction. The pellet was resuspended in the same buffer with 5% glycerol and dodecylmaltoside 1% and stirred for 1 h at 4 °C. The suspension was centrifuged at 50,000 rpm for 1h and the resulting supernatant contained the solubilized membrane proteins. The membrane protein extracts were used for either purification or electrophoresis.

#### 2.4.5. Visible Spectrophotometry of Purified Aer2so

Aer2so was purified from soluble proteins using a HisTrapFF column (GE Healthcare, UK) and the protein elution was performed in Tris-HCl pH 7.4 (40 mM) NaCl (300 mM) buffer and a 10 to 500 mM gradient of imidazole. Aer2so was eluted at 95 mM imidazole. It was then dialyzed against Tris-HCl pH 7.4 (40 mM) NaCl (50 mM) buffer overnight at 4 °C. Visible spectra of purified Aer2so were recorded from 350 nm to 700 nm using a Cary 60 UV-Vis (Agilent Technologies, Santa Clara, CA, USA) spectrophotometer. Dioxy-heme was done by adding dithionite to the sample.

#### 2.4.6. Blue Native Electrophoresis (BNE) Analysis and Immunodetection

Protein concentrations were determined using the Lowry modified method [40] using bovine serum albumin as a standard. To visualise the presence of complexes, proteins were separated by BNE according to the method of Schägger [41], using a 3–12% linear polyacrylamide gradient. Membrane proteins (in 50 mM Bis-Tris, pH 7.0, 5% glycerol, 750 mM ACA, and detergent) were supplemented with a 0.5% or 5% stock solution of Coomassie Blue G250 in 500 mM ACA to obtain a detergent/blue G ratio of 4/1 (g/g). Gels were run with a constant current (20 mA) for 3 h (Mini-Protean II; Biorad, Gemenos, France). The entire gels were run with the blue cathode buffer (50 mM tricine, 15 mM Bis-Tris, pH 7.0, and 0.02% Coomassie Blue G250). The anode buffer constituted 50 mM Bis-Tris, pH 7.0. The apparent molecular mass of proteins was estimated using the native molecular mass markers NativeMark (Invitrogen, USA). After electrophoresis, gels were stained with Coomassie blue R-250 or scanned using the Typhoon FLA9500 laser scanner (GE Healthcare, UK) with excitation at 473 nm and filter LPB (GE Healthcare, UK) when the overproduced proteins had a GFP-tag. To analyze Aer2-6His in complexes, immunoblotting experiments were carried out and proteins were transferred onto PVDF membranes (immobilon-E Millipore) using the Bio-Rad tank transfer system. Aer2so-6His was detected with an anti-6His polyclonal antibody (Thermo). After analysis by fluorescence visualization or immunoblotting experiment, protein bands were cut out from gels stained with Coomassie blue and stored at −20 °C before analysis by mass spectrometry. Control bands were cut out according to the Rf of the band of interest (retention factor or relative mobility, which corresponds to the distance migrated by a band divided by the distance migrated by the dye front) that allows comparison of the migration of a protein from gel to gel.

#### 2.4.7. Mass Spectrometry-Based Proteomic Analyses

Trypsin digestions were performed as well as LC-MSMS analyses (Liquid Chromatography coupled to tandem mass spectrometry) performed on a Q-Exactive plus mass spectrometer (ThermoFisher Scientific, Villebon sur Yvette, France), as previously described [42].

#### 2.4.8. Swimming Plate Assays

Cells were grown overnight on LB plates in the presence of chloramphenicol when appropriated and stabbed with a sterile toothpick on M63 minimal medium swim plates (0.3% agar) containing 1 mM IPTG or 0.2% arabinose for p33Tac or for pBad33 derivative plasmids, respectively. Swimming plates were incubated 4 days at 28 °C before imaging. RP437 and *S. oneidensis* strains were inoculated into tryptone soft agar containing 0 or 200 μM IPTG [43], and incubated at 20 °C for 16 h before imaging.

#### 2.4.9. Fluorescent Microscopy

For fluorescence microscopy, cells were grown aerobically overnight to reach stationary phase in aerated minimal medium at 28 °C. To test the effect of oxygen depletion on the dynamics of the clusters, after overnight growth under shaking, cells were submitted to an argon atmosphere. The microscopic plates were prepared in a glovebox and conserved under argon atmosphere until their use in the microscope. For all samples, 2 μL of living cells are spotted onto thin pads of PBS 1% agarose. To avoid the desiccation of the thin agar pads, the agar was poured onto squared adhesive frames previously pasted onto glass slides. The slides were directly observed and photographed with a Nikon TiE PFS inverted epifluorescence microscope (100 × oil objective NA 1.45 Phase Contrast) and a Hamamatsu Flash4 sCMOS camera. Images were collected with NIS elements software. Images analyses were performed with Fiji/Image J.

## 3. Results

### 3.1. MCPs and Chemosensory Systems in S. oneidensis

In order to better characterize the *S. oneidensis* chemosensory networks and assign each MCPs to one of the three Che systems, we decided to determine which class Che systems and MCPs belong to according to the MiST classification [44]. To do this, we took advantage of specific amino acid sequences important in *P. aeruginosa* CheA- or CheW-MCP interactions, depending on whether these proteins are part of Che systems of the F6 or F7 class [17] (Figure 1b). These sequences are conserved in *S. oneidensis* and allowed to first attribute the Che3 and Che1 to the F6 and F7 classes, respectively (Figure 1b). Che3 (F6) would be composed ofdan CheA3, CheW3 (SO_3202), and CheV (a CheW-CheY fusion, SO_3252). A second CheW-like (SO_3203) is also encoded by the *che3* locus but might not be associated with Che3. Indeed, conserved glutamate, normally forming a salt bridge with an arginine, as well as other CheW residues important in the interaction with MCPs, are absent in SO_3203. The Che2 locus belongs to the F8 class [44], but as mentioned above, might be non-functional or incomplete.

Next, we analyzed the products of the 24 orphan MCP-encoding genes scattered over the genome. From this analysis, it was possible to attribute 23 MCPs to the 40H class and the Che3 (F6) array. One of these MCPs, SO_3510 does not possess the conserved sequence for binding to CheW and thus cannot be attributed to any chemosensory system using this motif-based analysis. Three *S. oneidensis* MCPs are located in *che* clusters and can be classified as such. Firstly, *so_2323*, encoded by the *che2* locus and belonging to the 34H class, possesses no conserved motif that could link it to the Che1 (F7) or Che3 (F6) sensory pathway, alike SO_3510. SO_2117 and SO_2123 are encoded by the *che1* cluster. However, only SO_2123 is predicted to signal to Che1 (F7) while SO_2117 might be linked to Che3 (F6). This is consistent with what has already been established for their homologs in *P. aeruginosa* and *V. cholerae*. While all MCPs designated to signal to Che3 (F6) belong to the 40H class, SO_2117 is classified as a 44H-class MCP [44]. SO_2123 is a 36H MCP like its homologs in other bacteria. According to their homology with MCPs of *P. aeruginosa* and *V. cholerae*, we renamed SO_2117 and SO_2123, McpAso and Aer2so, respectively. While genes encoding McpA and Aer2 in *P. aeruginosa* are under the control of distinct regulations [10], the corresponding homologs in *S. oneidensis* seem to be co-transcribed. To verify this hypothesis, we performed RT-PCR experiments using appropriate primers annealing all along the *che1* locus (Figure 1c). As expected, given that the *che1*-locus genes are either overlapping or separated from each other by less than 22 bp, the analysis of the various amplification products indicates that *so_2117* and *so_2123* belong to a single transcriptional unit encompassing genes from *so_2117* to *so_2126* (Figure 1c). However, the presence of internal promoters leading to the synthesis of monocistronic mRNAs cannot be excluded. As mentioned above, it is noteworthy that McpAso and Aer2so, although belonging to the same transcriptional unit, are probably part of distinct pathways.

### 3.2. Aer2so Is Related to the Che1 Machinery

**Figure 2:**
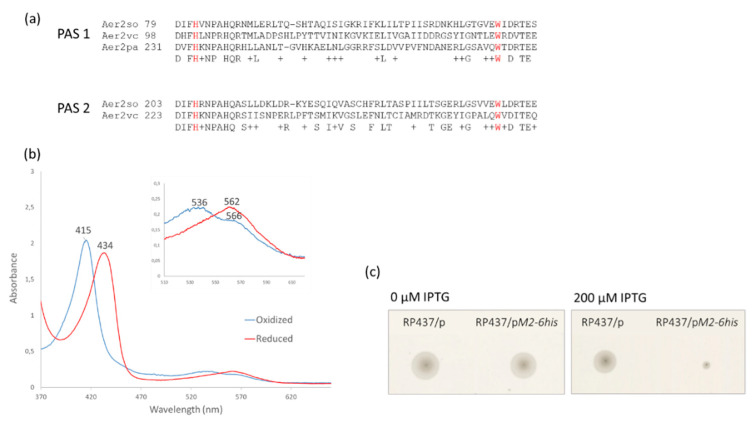
Aer2so characteristics. (**a**) Sequence alignment of Aer2 PAS domains of *S. oneidensis*, *V. cholerae*, and *P. aeruginosa* by Clustal Omega. The conserved histidine and tryptophan involved in heme coordination and O_2_-binding stabilization [48], respectively, are highlighted in red. “+” indicates similar amino acids. (**b**) Absorption spectra of purified Aer2so in the reduced and oxidized states. Aer2so was fully oxidized during purification and its reduction was obtained with dithionate. The wavelength for each absorbance maximum is indicated. An expanded view of peaks between 530 and 610 nm is shown in the inset. (**c**) Effect of Aer2so on *E. coli* chemotaxis. *E. coli* RP437 producing, or not producing, Aer2so-6his was inoculated in tryptone soft agar with 0 or 200 μM IPTG. The plates shown are representative of at least three independent experiments.

The Aer2so amino acid sequence shares 60% of its identity with that of *V. cholerae* Aer2 (Aer2vc) [45,46]. Aer2so and Aer2vc contain two PAS domains while Aer2pa (Aer2 from *P. aeruginosa*) contains one PAS domain. Each PAS domain of Aer2vc binds to a *b*-type heme. Some conserved residues such as a histidine were shown to be involved in heme coordination in Aer2pa and Aer2vc, and a tryptophan was shown to stabilize O_2_ in Aer2pa. Aer2so also contains these conserved histidine and tryptophan residues on its two PAS domains (Figure 2a). In order to purify Aer2so, we fused it to a 6His tag in C-term, produced it from *E. coli*, and purified it from solubilized membrane fractions. For the Aer2so-6His purification, we took advantage of the fact that its spectral profile can be followed in *E. coli* cell extracts due to the absence of production of cytochrome with similar spectra in this bacterium under the tested growth conditions [47]. The analysis of the redox spectrum obtained indicates that such a spectrum is characteristic of *b*-type hemes, judged by both a red-shifted Soret peak and a single broadband replacing the α/β bands in the reduced state, as observed for the two Aer2vc PAS domains [48] (Figure 2b). Finally, we tested whether Aer2so could inhibit *E. coli* chemotaxis alike its homolog Aer2vc [48]. Plasmid expressing *so_2123-6his* was introduced in *E. coli* RP437. Production of Aer2so significantly decreased *E. coli* RP437 swimming, as observed with Aer2vc indicating that the protein is functional and could compete with other *E. coli* MCPs for CheA binding or bind to *E. coli* MCPs, both cases confirming that Aer2so belongs to the 36H class (Figure 2c).

**Figure 3:**
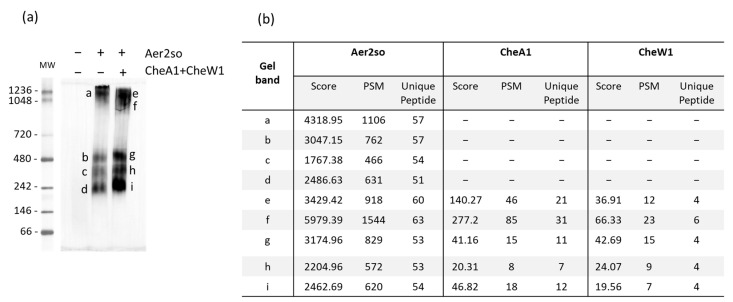
Aer2so forms core signaling units with CheA1 and CheW1. (**a**) Aer2so complexes visualized by BNE. Solubilized membranous extracts of Δ*cheA1* strain containing either p*M2-6his* or p*AWM2-6his* allowing the production of Aer2so or Aer2so, CheA1 and CheW1, respectively, were submitted to Blue Native Electrophoresis (BNE, 3–12%). Extracts of strain Δ*cheA1*/p33*Tac* were loaded as a control. Aer2so was detected by a western blot using a His-tag antibody. a to i correspond to the Aer2so containing bands analyzed by mass spectrometry. MW: molecular weight expressed in kDa. (**b**) Mass spectrometry identification of complex components. Table heading: Gel band, letters refer to the Blue-Native gel western (**a**) Score, protein score given by Sequest algorithm; PMS, number of peptide spectral matches given by the algorithm corresponding to the total number of identified peptide sequences for the protein, including those redundantly identified; and Unique peptides, number of distinct peptides matching to protein sequence and unique to this protein. The gel shown and the mass spectrometry analyses are representative of at least three independent experiments.

The bioinformatics analyses described above suggest that Aer2so signals to Che1 (F7). To validate this prediction, we co-expressed Aer2so with Che1 proteins in *E. coli* and checked for complex formation. Thus, to study the association of Aer2so to CheA1 and CheW1, we performed Blue Native Electrophoresis (BNE), a biochemical approach leading to the visualization of large-size complexes in cellular extracts. Native electrophoresis is a powerful technique that has been applied to identify functional complexes in eukaryotic and prokaryotic cells [49]. First, we expressed *so_2123-6his* from an *E. coli* strain devoid of its own MCPs (BT3388) to avoid interference since *E. coli* MCPs and Aer2so all belong to the same 36H class. The solubilized membranous fractions were submitted to BNE, and Aer2so was followed by a western blot using an anti-6His antibody. We observed that Aer2so formed high molecular weight complexes ranging from 220 to >1000 kDa (Figure 3a) indicating that the protein can oligomerize as expected for chemoreceptors since the Aer2so is stable and functional. Mass spectrometry results show that these Aer2so complexes are not associated with CheA or CheW from *E. coli* (Figure 3a, bands a–d). Although it was proposed that Aer2vc inhibits chemotaxis of *E. coli* by titrating chemotaxis components away from native MCPs [48], it seems that the swimming decrease of *E. coli* producing Aer2so is not due to titration of CheA or CheW. However, another explanation could be that the absence of membranous MCP in BT3388 leads to an unstable association of CheA and CheW with Aer2so.

To determine whether CheA1 and CheW1 interact with Aer2so, we co-expressed the *cheA1*, *cheW1*, and *so_2123-6his* genes in *E. coli* BT3388 and, as above, looked for complexes in solubilized membrane extracts. Several bands corresponding to high molecular weight complexes were detected in the membranous extracts (Figure 3a). Surprisingly, these complexes showed similar sizes to those obtained with Aer2so produced alone. The protein content of five bands was analyzed by mass spectrometry to identify the complex components and examined for the presence of CheA1 and CheW1. In all the submitted samples, Aer2so, CheA1, and CheW1 were detected (Figure 3b, bands e–i), suggesting that these three proteins all belong to Che1 (F7) arrays.

### 3.3. Che1 Forms Complexes In Vivo

**Figure 4:**
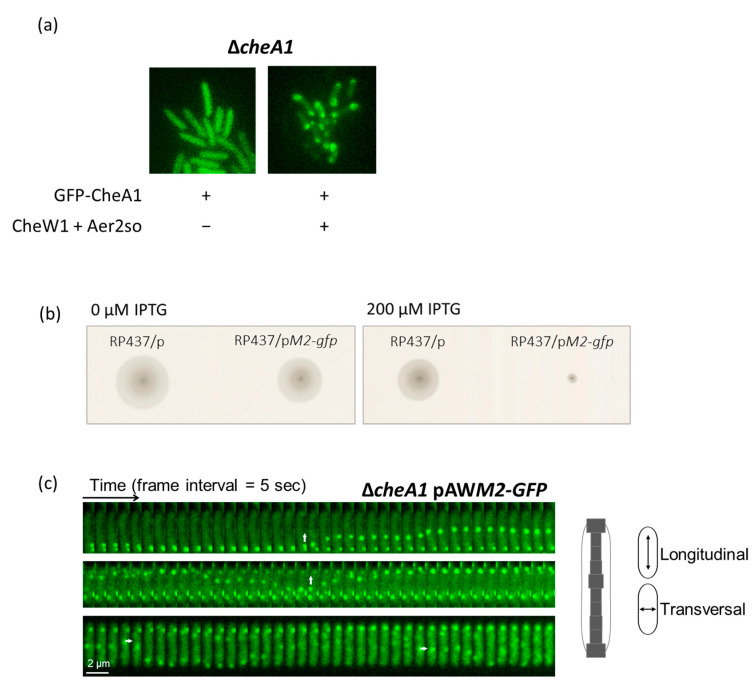
Subcellular localization of Che1 proteins in *S. oneidensis* and time-lapse recording. (**a**) Fluorescence images of the Δ*cheA1* strain containing either p*cheA1-GFP* or pGFP-*AWM2*, allowing the production of GFP-CheA1 alone or in complex with Aer2so and CheW1, respectively. Cells are from the stationary phase of growth in the minimum medium. Images are representative of at least three independent experiments. (**b**) Aer2so-GFP inhibits *E. coli* chemotaxis. *E. coli* RP437 producing, or not producing, Aer2so-GFP were inoculated in tryptone soft agar with 0 or 200 μM IPTG. The plates shown are representative of at least three independent experiments. (**c**) Fluorescence images of three representative cells showing the localization of Aer2so-GFP in a complex with CheA1 and CheW1, in the Δ*cheA1* strain. Cells are from the stationary phase of growth in a minimum medium. In total, 40 images were acquired with a frame interval of 5 s. The histograms of the fluorescent cluster distribution across the transversal axis cells are shown to the right of the fluorescence images. On the *x*-axis, the frequency of the clusters is represented and, on the *y*-axis, their relative position in the cell. White arrows show the longitudinal and transversal movements of the Aer2so-GFP clusters. The analysis was performed from three independent experiments.

We wanted to look at the localization of CheA1/CheW1/Aer2so complexes in living cells to define the localization of the Che1 (F7) array in *S. oneidensis*. For this, we used a cell biology approach. We constructed a Δ*cheA1 S. oneidensis* strain containing a plasmid harboring either *GFP-cheA1* (p*GFP-cheA1*) alone or with *cheW1* and *aer2so* (p*GFP-AWM2*). Recombinant cells were grown overnight to reach the stationary phase in an aerated minimal medium. GFP-CheA1 stability was confirmed by western blotting (Figure S2). To visualize GFP-CheA1 fluorescence, cells were spotted on a thin layer of agarose and images were captured every 5 s for 4 min by time-lapse fluorescence microscopy. When CheA1-GFP was produced alone, the fluorescence signal was diffused throughout the cells alike that of CheA2 from *P. aeruginosa* [50] (Figure 4a). Contrarily, in the presence of Aer2so and CheW1 produced from expression of p*GFP-AWM2*, complexes were detected equally located at both poles of the cell with discrete foci present in the centre of the cell showing that the presence of Aer2so and CheW is required to form CheA1-containing clusters (Figure 4a, Figure S3 and Video S3). This indicates that when GFP-CheA1 was produced from the plasmid expression, and CheW1 and Aer2so were produced from chromosomal expression, either the stoichiometry of the three proteins did not permit complex formation or a possible polar effect of the cheA1 mutation affected the expression of downstream genes. The results obtained indicate that CheA1 forms a complex with Aer2so correctly localized in the cell, although there is no phenotype associated with CheA1 to control whether the recombinant protein retains behaviors of the native protein. Interestingly, like Aer2so-6his, Aer2so-GFP hampers *E. coli* RP437 swimming indicating that GFP does not modify its behaviors (Figure 4b). Therefore, we fused the Aer2so gene to the GFP gene in a plasmid and looked for the Aer2so-GFP subcellular localization. Aer2so-GFP, CheA1, and CheW1 were produced in *S. oneidensis* from the expression of p*AWM2-GFP*. To visualize Aer2so-GFP in the presence of CheA1 and CheW1, cells were treated as described above. We observed that Aer2so-GFP was produced and formed fluorescent clusters at the cell poles but also all along the cell (Figure 4c). Surprisingly, these clusters were found to be dynamic, continuously changing their position within the living cells. Their motion followed two distinct patterns: one along the longitudinal axis from one cell pole to the other, and the second, around a transversal axis. Moreover, clusters seemed to flicker when localized along the transversal axis (Figure 4c, Video S4). To understand why the patterns were different according to the GFP-fused protein, we looked at Aer2so-GFP produced alone from p*M2-GFP* expression. Once again, cells were spotted on a thin layer of agarose and images were captured every 5 s for 4 min by time-lapse fluorescence microscopy. Similar dynamic fluorescent clusters as those observed in the presence of CheA1 and CheW1 were observed (Video S5). These fluorescent clusters correspond to the Aer2so complexes as detected by BNE (Figure 3a), since when Aer2so-GFP is produced in *E. coli* cells lacking their own MCPs, both clustering and dynamics of Aer2so are detected (Video S6). This alsosuggests that no other *S. oneidensis* specific protein is required for Aer2so clustering and localization. Altogether, these results indicate that in the presence of CheA1 and CheW1, fluorescent clusters are heterogeneous in their composition, with some containing Aer2so alone and others containing Aer2so in a complex with CheA1 and CheW1, as shown by the BNE approach. Next, we wanted to determine whether the cell growth phase could affect the localization and dynamics of the Aer2so-GFP fluorescent foci. For this, we compared fluorescent micrographs of cells in the stationary phase with that of cells in the exponential growth phase (Video S7). The dynamics of the foci were similar, as observed above. Since Aer2 from other organisms was shown to sense and bind oxygen, we were interested in determining whether a rapid depletion of O_2_ could affect the dynamics of Aer2so-GFP clusters. Therefore, after overnight growth under shaking, cells were kept under an argon atmosphere in order to deplete the oxygen. We observed that the Aer2so clusters were still dynamic and localized around the membrane (Video S8).

### 3.4. McpAso Forms Clusters with CheA3

**Figure 5:**
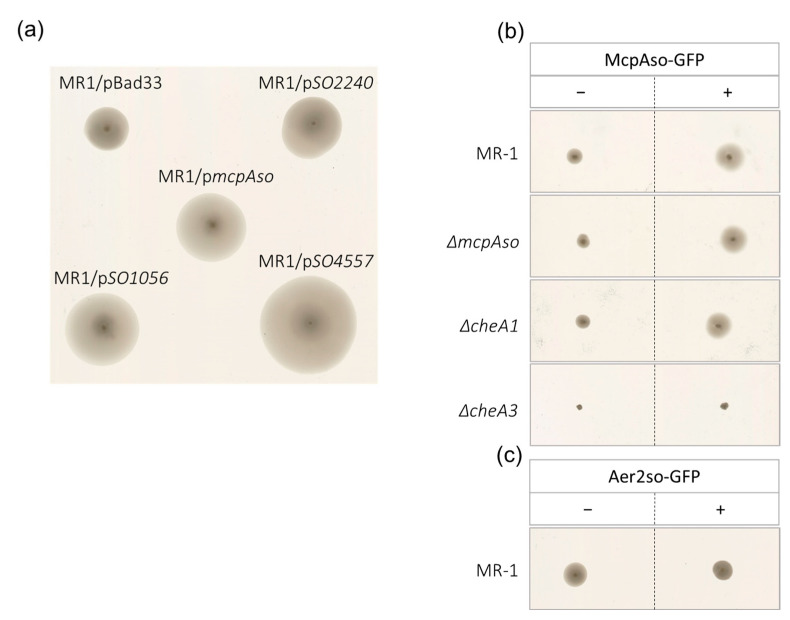
McpAso overproduction increases *S. oneidensis* swimming. (**a**) Wild type *S. oneidensis* strain (MR1) containing a vector (pBad33) or a plasmid allowing the overproduction of McpAso or other MCPs (SO_4557, SO_1056, and SO_2240) were grown on LB plates and stabbed on minimum medium soft agar plates with 0.2% arabinose. (**b**) Wild type *S. oneidensis* strain (MR1) or mutant derivatives (Δ*mcpAso*, Δ*cheA1*, and Δ*cheA3*) containing either the empty vector p33*Tac* (−) or a plasmid allowing the overproduction of McpAso-GFP (+) were grown overnight on LB plates and stabbed on minimum medium soft agar plates with 1mM IPTG. (**c**) Alike (**b**), except with respect to the wild type *S. oneidensis* strain (MR1) containing p*M2-GFP* (+) allowing the overproduction of Aer2so-GFP.

To determine McpAso cell localization, a McpAso-GFP fusion was constructed and expressed from a plasmid transferred into wild type *S. oneidensis* cells as well as into various *che1* and *che3* mutants. A hint that McpAso-GFP is functional is that it enhances *S. oneidensis* swimming on a soft minimal medium when overexpressed, similarly to McpAso without GFP (Figure 5a,b). As controls, three other MCPs of the 40H class available in the laboratory were tested. In total, two of them, SO_1056 (chromium and malate attraction, nickel, and cobalt repulsion) [39,51] and SO_2240 (aerotaxis) [32], were proved to be involved in chemotaxis. The third, SO_4557, has yet unknown functions. The overproduction of SO_1056, SO_4557, and SO_2240 led to increased swimming as observed for McpAso, meaning that this approach could be informative of the involvement of an MCP in chemotaxis (Figure 5a). Aer2so was also tested under the same conditions, and no modification of the swimming behavior was observed (Figure 5c).

Plates were incubated at 28 °C for four days. Results shown on the plates are representative of at least three independent experiments.

**Figure 6:**
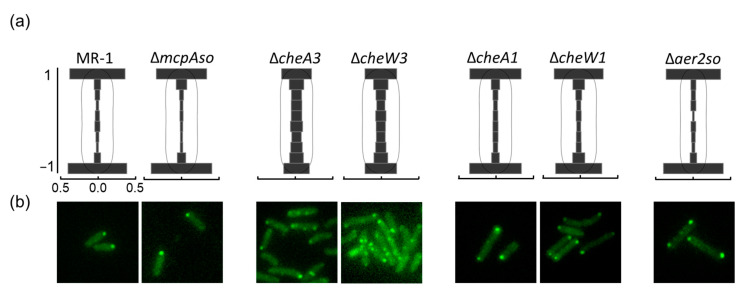
McpAso localization in various *S. oneidensis* mutants. (**a**) Histograms of the fluorescent cluster distribution across the transversal axis cells in the different mutants are shown. On the *x*-axis, the frequency of the clusters is represented and, on the *y*-axis, their relative position in the cell. (**b**) Fluorescence images of the various *S. oneidensis* mutants in the stationary phase of growth in a minimum medium are represented. The analysis was performed from three independent experiments.

To further confirm the association of McpAso to the Che3 chemotaxis system, strains overproducing McpAso and deleted of genes encoding either McpAso, CheA1, or CheA3 were tested. As expected, the absence of CheA3 abolished swimming while the absence of CheA1 did not affect the increased swimming due to the McpAso overproduction. Finally, the absence of McpAso did not affect the swimming capacity of *S. oneidensis* (Figure 5b). This is in accordance with McpAso driving cell mobility via Che3. Then, McpAso localization was studied in living cells. While McpAso-GFP formed one or two polar clusters when expressed in wild type, in the absence of either CheA3 or CheW3, McpAso-GFP formed multiple faint clusters at the periphery of cells (Figure 6). In contrast, the polar localization of McpAso-GFP was unchanged in the absence of either CheA1 or CheW1 (Figure 6). Finally, the absence of Aer2so did not modify the localization of McpAso-GFP at the cell pole. These results indicate that, as in *V. cholerae* and *P. aeruginosa*, McpAso efficient clustering at the cell pole depends on the presence of CheA3 and CheW3 and is independent of the Che1 machinery.

**Figure 7:**
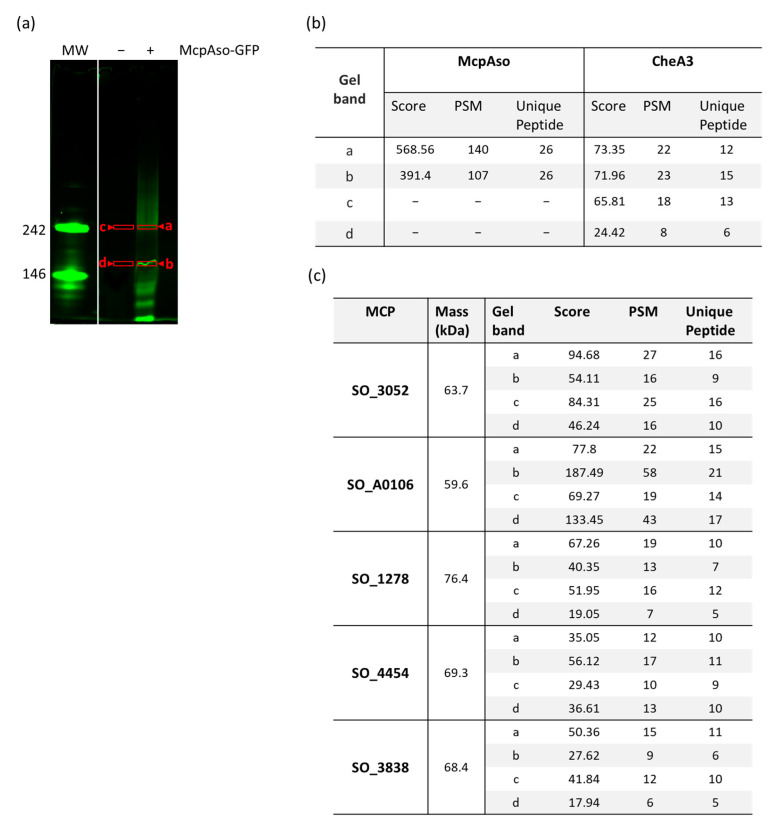
McpAso forms complexes with CheA3. (**a**) Detection of McpAso-GFP containing complexes in membrane extracts. Membrane extracts (30 μg) of strain Δ*mcpAso* containing (+), or not containing (−), p*mcpAso-GFP* that allows the production of recombinant McpAso-GFP were submitted to Blue Native Electrophoresis (BNE, 3–12%). Complexes were detected by fluorescence. a, b, c, and d indicate the bands that were cut out of the gel and analyzed by mass spectrometry. MW: molecular weight expressed in kDa. (**b**) Mass spectrometry identification of complex components. Table heading: Gel band, letters refer to the Blue-Native gel western. (**a**) Score, protein score given by Sequest algorithm; PMS, number of peptide spectral matches given by the algorithm corresponding to the total number of identified peptide sequences for the protein, including those redundantly identified; and Unique peptides, number of distinct peptides matching to protein sequence and unique to this protein. (**c**) Identification of other MCPs present in the complexes analyzed by mass spectrometry. Table heading as in (**b**) except mass, theoretical molecular mass of the identified protein given by the Sequest algorithm. The gel shown and the mass spectrometry analyses are representative of at least three independent experiments.

Next, to further characterize the association of McpAso with CheA3 and CheW3, complex formation with these proteins was analyzed by the BNE approach with mass spectrometry identification. For this, strain Δ*mcpAso*/p*mcpAso-GFP* was grown on a minimal medium and expression of *so_2117* from the plasmid was induced with IPTG overnight. Membrane extracts were prepared and solubilized prior to being submitted to BNE to determine McpAso partners. The expected size for each core-signaling unit composed of 6McpAso-GFP:2CheA3:2CheW3 was 600 kDa. However, a smear of fluorescence was observed between 242 to 600 kDa in tracks containing McpAso-GFP, with the highest band observed at about 240 kDa (Figure 7). This indicates that either the 6McpAso-GFP:2CheA3:2CheW3 complex was not stable during the membrane preparation, or the sensitivity of the fluorescence detection was not enough. In total, two of the visible bands (of about 146 and 242 kDa) were extracted and their content was analyzed by mass spectrometry (Figure 7a,b). As a control, bands at the same Rf were cut from samples in which McpAso-GFP was not produced and were also analyzed by mass spectrometry (Figure 7a,b). As expected, for samples of strain Δ*mcpAso*/p*mcpAso-GFP*, McpAso-GFP and CheA3 were identified (Figure 7b). CheW3 was not detected in our experiments due to either a weak detection by mass spectrometry or a loss during the membrane preparation. These results suggest that McpAso-GFP is part of core-signaling units with CheA3. In these experiments, additional MCPs belonging to the 40H class were also identified in the same bands with five of them present in each cut band (Figure 7c). As expected, these MCPs and CheA3 were also found in the control tracks (Figure 7b,c).

## 4. Discussion

In the *S. oneidensis* MR1 genome, three chemosensory gene clusters (*che1*, *che2*, and *che3*) have been identified. The Che3 system is involved in chemotaxis [22,24] while the Che1 could be involved in the posttranslational regulation of RpoS [28]. Two MCPs are genetically linked to Che1 since their genes are in the same transcription unit as the *che1* genes. These two MCPs, McpAso and Aer2so, are homologs of chemoreceptors found in *P. aeruginosa* and *V. cholerae*. Chemoreceptors, the CheA histidine kinase, and the CheW connecting protein, form core-signaling units that assemble to build arrays that transduce environmental signals allowing a rapid response of the bacteria for survival as chemotaxis, biofilm formation, and virulence [1]. Based on consensus motifs established in *P. aeruginosa* [17], we propose that also in *S. oneidensis* MCP could be assigned to one or the other chemosensory systems. Moreover, in this study, we have confirmed experimentally that Aer2so is connected to the Che1 system whereas McpAso might form core-signaling units with the Che3 system. 

CryoET experiments have shown that beside the F6 array, an unusually tall F7 array that lacks periplasmic density was present in *V. cholerae* and *P. aeruginosa* [18]. In these organisms, the absence of Aer2, CheA, or CheW associated with this F7 pathway lead specifically to the loss of arrays. It was also proposed that the tall F7 array observed in *S. oneidensis* included Aer2so. We have shown that when Aer2so, CheA1, and CheW1 were co-produced, the three proteins were present in the same complexes. In vivo, these complexes were localized mainly at the two cell poles. It seems that some clusters are also visible in the centre of the cell. It was proposed that this distribution facilitates the repartition of the chemosensory arrays during cell division allowing each daughter cell to inherit functional arrays and adapt rapidly to the environment without having to wait for de novo synthesis [52]. Interestingly, Aer2so forms dynamic complexes distributed along the longitudinal and transversal axis of the cell even in the absence of CheA1 and CheW1. Contrary to McpAso, which appears to be static, Aer2so assembles into complexes that continuously change position. We observed that a rapid decrease of O_2_ availability described as Aer2so signal did not hamper Aer2so dynamics, nor did the growth phase tested in this study. Several hypotheses can be proposed concerning the Aer2so dynamics and clustering. Indeed, this organization can, for instance, enhance the probability to form core-signaling units with CheA1 dimers and CheW1 in the case of stochastic self-assembly of the F7 array or/and ensure a correct repartition between progeny on cell division [52,53]. It is noteworthy that according to their genetic organization, the Che1 system (including Aer2so) and McpAso are probably involved in RpoS posttranslational regulation and thus have an essential role in the global stress response of *S. oneidensis*. Therefore, it could be important for the cell to keep a “basal” level of the F7 array even during cell division. It could be interesting to check whether Aer2so homologs in *P. aeruginosa* and *V. cholerae* share the same behaviors. An MCP from *Myxococcus xanthus*, FrzCD, is cytoplasmic and forms dynamic clusters [54]. However, FrzCD clusters are evenly distributed along the nucleoid while Aer2so clusters present a distribution along the longitudinal and transversal axis of the cell. Finally, since Aer2so and FrzCD are both cytoplasmic, we wonder if the MCP dynamic clustering is specifically linked to F7 arrays and/or to cytoplasmic MCPs. 

Although it was proposed that Aer2vc inhibits chemotaxis of *E. coli* by titrating chemotaxis components away from native MCPs [48], Aer2so has not been found in complex with *E. coli* CheA. One possibility is that CheA and CheW do not stably associate with a cytoplasmic Aer2so in the absence of any other transmembrane chemoreceptor. Another hypothesis could be that the swimming decrease of *E. coli* producing Aer2so is not due to titration of CheA or CheW. Indeed, it is noteworthy that the conserved sequence of *E. coli* CheA involved in MCPs binding (LNAVMES) differs from that of CheA1. Our hypothesis is that Aer2so could titrate the native *E. coli* chemoreceptors that belong to the same 36H class forming heterologous clusters of chemoreceptors. 

The absence of CheA3 leading to a diffuse distribution of McpAso and the presence of McpAso in complexes containing CheA3 indicate that this MCP might form core-signaling units with CheA3. Thus, McpAso is part of the chemotaxis machinery of *S. oneidensis* forming F6 arrays at mainly one pole of the cell. Although 23 other MCPs produced by *S. oneidensis* might also signal to CheA3 (F6), McpAso belongs to a different class of MCPs. However, the mass spectrometry analysis suggests that McpAso is found in complexes with other MCPs and CheA3. Since core-signaling units can contain distinct dimers of MCPs as far as they belong to the same heptad class, this point raises the question of whether McpAso can be part of core-signaling units with other MCPs. The heptad number rule suggests that McpAso does not mix with other MCPs [55]. However, sincr McpAso was unclassified or sometimes classified as 44H, it is thus possible that it forms specific core-signaling units in a chemosensory array containing core sensory units composed by the other detected chemoreceptors. This means that core-signaling units of various classes can be part of the same array. Nevertheless, the heptad number of McpAso and homologs seems difficult to assess and one can imagine that McpAso could fit with the 40H class MCPs. These results confirm the involvement of McpAso in chemotaxis, likely forming specific core-signaling units among the other containing 40H type MCPs, altogether building a chemotaxis array at the cell pole of the bacterium. 

In conclusion, McpAso and Aer2so are two chemosensory receptors whose genes belong to the same operon, but which are part of two distinct Che systems. While McpAso is dedicated to chemotaxis with the Che3 system, Aer2so is probably involved in the global stress response with the Che1 system. Lately, new knowledge in the field of chemosensory systems is developing rapidly. For example, *Comamonas testosteroni* has two gene clusters encoding chemosensory pathways and it was shown that a cross talk between them might coordinate chemotaxis and biofilm formation [56]. In *Azospirillum brasilense*, mixed arrays comprising proteins of distinct chemotaxis systems and chemoreceptors of distinct families allow signal integration and coordinated response output [20]. We do not know yet whether it could be similar in *S. oneidensis*. However, our results indicate that the two MCPs we characterized interconnect the Che1 and Che3 systems allowing bacterial survival through motility to seek better environments and global stress response to adapt.

## Figures and Tables

**Table 1:** Strains used in this study.

Strains	Characteristic and Genotypes	References
*S. oneidensis* strains		
MR1-R	*Shewanella oneidensis*, wild-type strain, Rif^R^	[31]
Δ*cheA1*	MR1-R *deleted of cheA1 (so_2121)*	[22]
Δ*cheW1*	MR1-R deleted of *cheW1 (so_2122)*	This work
Δ*so2123* (Δ*aer2so*)	MR1-R deleted of *aer2so (so_2123)*	[32]
Δ*mcpAso*	MR1-R deleted of *mcpAso (so_2117)*	This work
Δ*cheA3*	MR1-R deleted of *cheA3 (so_3207)*	[22]
Δ*cheW3*	MR1-R deleted of *cheW3 (so_3202)*	[22]
*E. coli* strains		
CC118 *λpir*	Δ*(ara-leu) araDE* Δ*lacX74 galE galK phoA20 thi-1 rpsE rpoB argE (Am) recA1 λpir*	[33]
C600	*F- tonA21 thi-1 thr-1 leuB6 lacY1 glnV44 rfbC1 fhuA1 λ-*	[34]
RP437	*F- thi thr leu his met eda rpsL, wild type for chemotaxis*	[35]
BT3388	*E. coli* strains RP437 (wild type) deleted of *tar, tsr, trg, tap, aer*	[36]

**Table 2:** Plasmids used in this study.

Plasmids	Descriptions	References
pKNG101	R6K-derived suicide plasmid containing Str^R^ and *sacB*	[33]
pBad33	Vector containing pBAD promoter with the pACYC origin of replication	[38]
p33Tac	Derived vector of pBad33 containing ptac promoter inducible to IPTG	[29]
p*M2-6his*	p33Tac containing sequence coding for C-terminal 6His-tagged Aer2so	This work
p*M2-GFP*	Sequence coding for Aer2so-GFP cloned into p33Tac	This work
p*AWM2-6his*	*cheA1-cheW1-aer2so*-*6his* sequences cloned into p33Tac	This work
p*AWM2-GFP*	*cheA1-cheW1-aer2so*-*GFP* sequences cloned into p33Tac	This work
p*GFP-cheA1*	*GFP-cheA1* sequence cloned into p33tac	This work
p*GFP-AWM2*	*GFP-cheA1-cheW1-aer2so* sequences cloned into p33Tac	This work
p*mcpAso-GFP*	*so2117-GFP* sequence cloned into p33Tac	This work
p*SO4557*	*so_4557* sequence cloned into pBad33	[39]
p*SO2240*	*so_2240* sequence cloned into pBad33	[39]
p*SO1056*	*so_1056* sequence cloned into pBad33	[39]
p*mcpAso*	*so_2117 sequence cloned into pBad33*	[32]

## Data Availability

Not applicable.

## Supplementary Materials

The following supporting information can be downloaded at: https://www.mdpi.com/article/10.3390/biom13010021/s1, Figure S1: Scheme summarizing the proteins of CheA, CheW, and MCPs (including Aer2so and McpA) of Che1 and Che3 chemosensory arrays of *S. oneidensis*; Figure S2: CheA1-GFP stability; Figure S3 and Video S1: Localization of GFP-CheA1 co-produced with CheW1 and Aer2so; Video S2: Aer2so-GFP co-produced with CheA1 and CheW1 in Δ*cheA1 S. oneidensis*; Video S3: Aer2so-GFP clusters in Δ*cheA1 S. oneidensis*; Video S4: Aer2so-GFP clusters with CheA1 and CheW1 in BT3388 *E. coli* strain; Video S5: Aer2so-GFP co-produced with CheA1 and CheW1 in Δ*cheA1 S. oneidensis* strain during exponential growth; Video S6: Aer2so-GFP co-produced with CheA1 and CheW1 in Δ*cheA1 S. oneidensis* after oxygen depletion.
