# Supplementary material for: Defining Two Chemosensory Arrays in Shewanella oneidensis
Source: Biomolecules. 2022 Dec 22;13(1):21. doi: 10.3390/biom13010021 (PMC9855816; doi:10.3390/biom13010021)
Supplement: Supplementary file 1 [file biomolecules-13-00021-s001.zip › biomolecules-2100984-supplementary.pdf]

Defining two chemosensory arrays in *Shewanella oneidensis*

Emma M. Fortier [1], Sophie Bouillet[1,2], Pascale Infossi[1], Amine Ali Chaouche[1], Leon Espinosa[3], Marie-Thérèse Giudici-Orticoni[1], Emilia M.F. Mauriello[3], Chantal Iobbi-Nivol[1*]

Aix-Marseille Université, [1]Laboratoire de Bioénergétique et Ingénierie des Protéines, [2]Present address: NIH, NCI, 37, Convent Drive, Bethesda, MD 20814, [3]Laboratoire de Chimie Bactérienne, Institut de Microbiologie de la Méditerranée, Centre National de la Recherche Scientifique, 13402 Marseille, France. * Corresponding author: iobbi@imm.cnrs.fr

Supplemental information.

Methods

List of primers used in Figure 1

a	TTTGAAAACCCCTGAATCTC
b	GAGTAGATTTTTCATCAGCACATTG
c	CATCAGCCCATGAACTTGAA
d	CCCATGCATTCCCTTTACTG
e	CACGTTATAACCTTTTCCGTGA
f	TGCCTGCGGTTCTTTCTTAT
g	GATCAAAATATGCCACGGATG
h	TGATGGGAGTCCGCATAAAT
i	TTTGAAGAAAGCCATGAGCA
j	TTCTTCAGGATCTGGCGACT
k	GGGTAACCCCCAAACCTAAA
l	ACACGCACATTACCTTGCTG
m	TTGCGCTTAATGAGCCAAAT
n	GCGGATTAATGTGGAATCGT

Fluorescence microscopy

For fluorescence microscopy, cells were grown aerobically overnight to reach stationary phase in aerated minimal medium at 28°C. For all samples, 2 µL of living cells are spotted onto thin pads of PBS 1 % agarose. To avoid the desiccation of the thin agar pads, the agar was poured onto squared adhesive frames previously pasted on to glass slides. The slides were directly observed and photographed with a Nikon TiE PFS inverted epifluorescence microscope (100 x oil objective NA 1.45 Phase Contrast) and a Hamamatsu Flash4 sCMOS camera. Images were collected with NIS elements software. Images analysis were performed with Fiji/ Image J.

Video S4 As above except that cells were observed during exponential growth phase.

Video S5 as for videos S1 except that we test the effect of oxygen depletion on the dynamic of the clusters, after overnight growth under shaking, cells were submitted to argon atmosphere. The microscopic plates were prepared in glovebox and conserved under argon atmosphere until their use in the microscope.

Figure S1 : Scheme summarizing the proteins of CheA, CheW and MCPs (including Aer2so and McpA) of Che1 and Che3 chemosensory arrays of *S. oneidensis*.

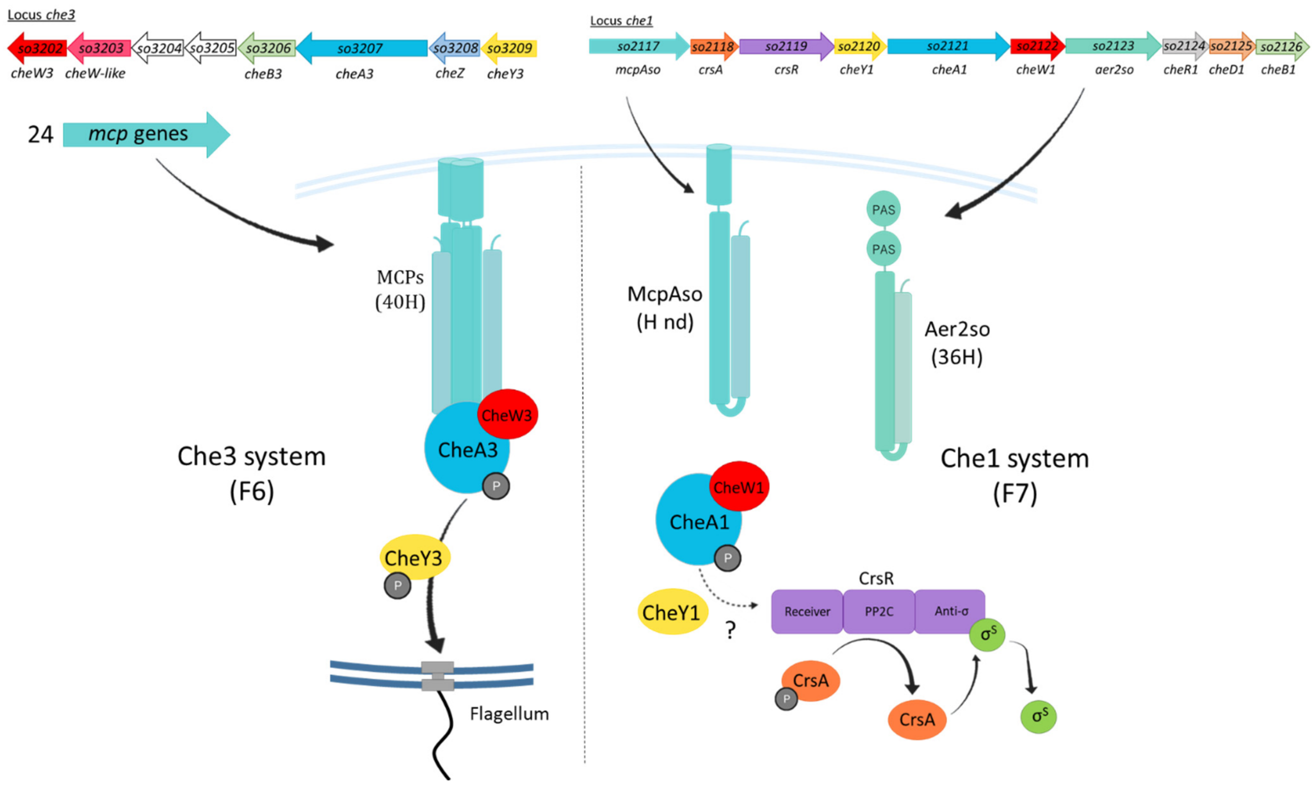

(H nd) indicates that the heptad number of McpAso is not clearly defined.

Figure S2 : CheA1-GFP stability

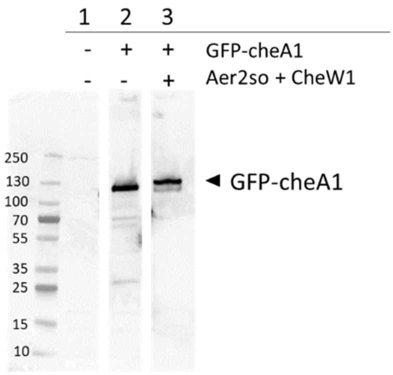

Δ*cheA1* containing either, p33tac (1), p*GFP-cheA1* (2) or p*GFP-AWM2* (3) were submitted to SDS-PAGE and GFP-CheA1 was detected by Western blotting using a GFP tag antibody.

Figure S3 and Video S1: **Localization of GFP-CheA1 co-produced with CheW1 and Aer2so.**

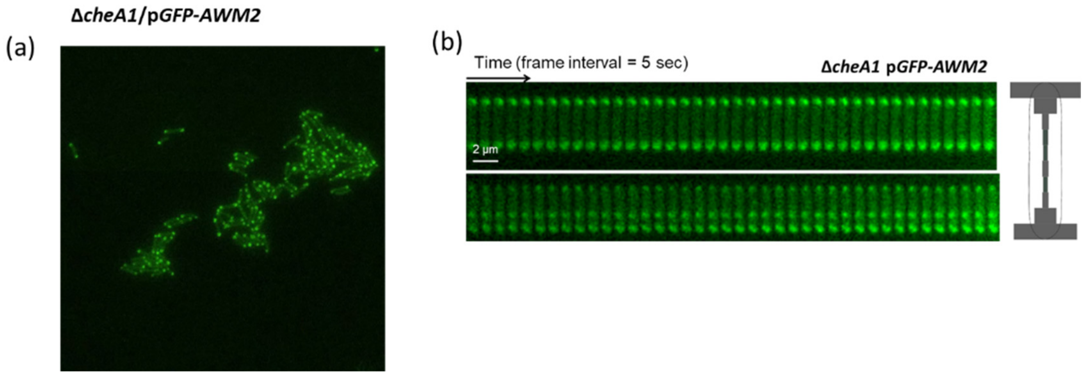

(a) Localization of GFP-CheA1 in presence of Aer2so and CheW1 in Δ*cheA1 S. oneidensis*.

dcheA1 pGFP-cheA1-cheW1-mcp2.avi

(b) Fluorescence images of 2 representative cells for localization of Che1 complexes with GFP-labeled CheA1, in Δ*cheA1* strain. 40 images were acquired with a frame interval of 5 seconds. The histograms of the fluorescent clusters distribution across the transversal axis cells are shown to the right of the fluorescence images. On the x-axis the frequency of the clusters is represented and on the y-axis their relative position in the cell. The analysis was performed from three independent experiments.

Video S2 : Aer2so-GFP co-produced with CheA1 and CheW1 in Δ*cheA1 S. oneidensis*.

dcheA1 pAWM2-GFP.avi

ΔcheA1/pAWM2-GFP

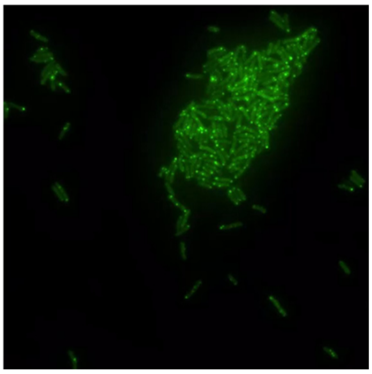

Video S3 : Aer2so-GFP clusters in Δ*cheA1 S. oneidensis*.

dcheA1pM2-GFP.avi

ΔcheA1/pM2-GFP

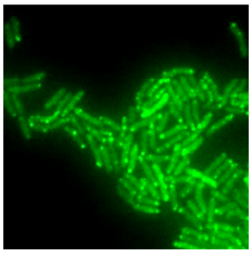

Video S4 : Aer2so-GFP clusters with CheA1 and CheW1 in BT3388 *E. coli* strain.

BT3388 pAWM2-GFP.avi

BT3388/p*AWM2-GFP*

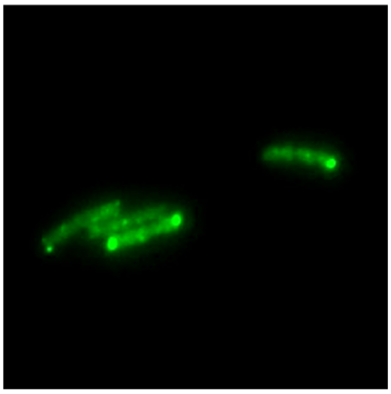

Video S5 : Aer2so-GFP co-produced with CheA1 and CheW1 in *ΔcheA1 S. oneidensis* strain during exponential growth.

dcheA1 pAWM2-GFP exponential growth.avi

ΔcheA1/pAWM2-GFP

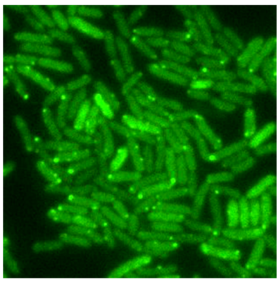

Video S6 : Aer2so-GFP co-produced with CheA1 and CheW1 in *ΔcheA1 S. oneidensis* after oxygen depletion.

dcheA1 pAWM2-GFP-O2.avi

ΔcheA1/pAWM2-GFP

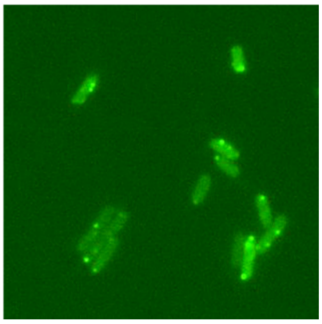

Legend of videos

Δ*cheA1 S. oneidensis* or BT3388 *E. coli* strains containing the plasmid indicated on the picture were grown overnight to reach stationary phase in aerated minimal medium (videos S2, S3, S4, S5). S6 as above except that cells were observed during exponential growth phase. S7 as for S3 except that cells were submitted to O_2 depletion. To visualize fluorescent proteins, cells were spotted on a thin layer of agarose and images captured every 5 s for 4 min by time-lapse fluorescence microscopy. 40 images were acquired with a frame interval of 5 seconds.
